# Combination of Hepatitis B Virus Pre-S2 Gene Deletion Mutation and Tumor-Node-Metastasis Stage Predicts Higher Hepatocellular Carcinoma Recurrence after Curative Surgical Resection

**DOI:** 10.3390/biomedicines11030923

**Published:** 2023-03-16

**Authors:** Long-Bin Jeng, Tsai-Chung Li, Wen-Ling Chan, Chiao-Fang Teng

**Affiliations:** 1 Organ Transplantation Center, China Medical University Hospital, Taichung 404, Taiwan; 2 Department of Surgery, China Medical University Hospital, Taichung 404, Taiwan; 3 Cell Therapy Center, China Medical University Hospital, Taichung 404, Taiwan; 4 Department of Public Health, College of Public Health, China Medical University, Taichung 404, Taiwan; 5 Department of Healthcare Administration, College of Medical and Health Science, Asia University, Taichung 413, Taiwan; 6 Department of Bioinformatics and Medical Engineering, Asia University, Taichung 413, Taiwan; 7 Epigenome Research Center, China Medical University Hospital, Taichung 404, Taiwan; 8 Graduate Institute of Biomedical Sciences, China Medical University, Taichung 404, Taiwan; 9 Program for Cancer Biology and Drug Development, China Medical University, Taichung 404, Taiwan; 10 Research Center for Cancer Biology, China Medical University, Taichung 404, Taiwan

**Keywords:** hepatocellular carcinoma, hepatitis B virus, pre-S2 gene deletion mutation, tumor-node-metastasis stage, recurrence

## Abstract

Hepatocellular carcinoma (HCC) is one of the most frequent and life-threatening human cancers worldwide. Despite curative resection surgery, the high recurrence rate of HCC leads to poor patient survival. Chronic hepatitis B virus (HBV) infection is a major etiological factor for HCC. HBV pre-S2 gene deletion mutation leads to the expression of an important oncoprotein called a pre-S2 mutant. It represents an independent prognostic biomarker for HCC recurrence. This study aimed to identify other independent prognostic biomarkers from clinicopathological characteristics of 75 HBV-related HCC patients receiving resection surgery and to validate their potential to be combined with pre-S2 gene deletion mutation as a combination biomarker for HCC recurrence. Patients with both the presence of pre-S2 gene deletion mutation and tumor-node-metastasis (TNM) stage IIIA–IIIC had a higher HCC recurrence risk than patients with either one or none of these two factors. Moreover, the combination of pre-S2 gene deletion mutation and TNM stage exhibited better performance than either of these two factors alone in discriminating patients from patients without HCC recurrence. Collectively, this study proposed that the TNM stage held significance as a combination biomarker with pre-S2 gene deletion mutation with a greater performance in predicting HCC recurrence after curative surgical resection.

## 1. Introduction

As the most common histological type of liver cancer, hepatocellular carcinoma (HCC) ranks as the sixth most prevalent human cancer and the third leading cause of cancer-related death worldwide, leading to over 800,000 new cases and 800,000 new deaths every year [1,2]. Although curative surgical therapies such as liver transplantation and resection surgery have been well established for treating HCC patients, the scarcity of available donor livers and high rate of postoperative HCC recurrence (up to 70% within five years of operation) largely limited their survival benefit for patients, respectively [3,4]. In addition, various locoregional therapies have been available as potential downstaging/bridging treatments for HCC. However, their therapeutic efficacy varies among patients and depends critically on patient selection [5,6]. Moreover, the existing systemic therapies such as chemotherapy and molecular targeted therapy are greatly challenged by high levels of drug resistance and genomic heterogeneity, resulting in poor outcomes for patients after treatment [7,8]. Therefore, it remains an important goal to develop biomarkers for effectively selecting the patients with a higher risk of HCC occurrence and recurrence for early detection and timely treatment to improve their prognosis.

Chronic infection with the hepatitis B virus (HBV) is one of the most significant risk factors for HCC. It is responsible for as high as 50% of total HCC cases worldwide [9,10]. The DNA genome of HBV contains four overlapping open reading frames (ORFs) including polymerase (P), core (C), surface (S), and (X), among which the S ORF consists of pre-S1, pre-S2, and S surface genes and expresses three different-sized surface proteins (small, middle, and large) from the S gene, the pre-S2 and S genes, and all three genes, respectively [11,12]. Several deletion mutations which naturally occur in the pre-S2 gene of the HBV DNA genome have been discovered and result in the expression of a mutation type of HBV large surface protein, called HBV pre-S2 mutant [13,14]. It has been well demonstrated that pre-S2 mutant functions as an HBV oncoprotein which plays a critical role in promoting HCC development through initiating multiple oncogenic signaling pathways to enhance the malignant phenotypes of hepatocytes in vitro and in vivo [15,16]. The pre-S2 mutant-activated oncogenic protein signals have been also validated as potential molecular drug targets for the chemoprevention of pre-S2 mutant-related HCC development in vivo [17,18,19]. Moreover, the presence of HBV pre-S2 gene deletion mutation in liver tissues or blood samples has been validated as a valuable independent biomarker for predicting a higher risk of HCC occurrence in patients with chronic HBV infection and HCC recurrence in patients with HBV-related HCC after curative surgical resection [20,21,22,23,24,25,26,27,28]. Therefore, it has a great promise to discover other independent biomarkers to be combined with pre-S2 gene deletion mutation for developing a combination biomarker with superior performance in predicting HBV-related HCC occurrence and recurrence.

In this study, many different clinicopathological characteristics obtained from a cohort of 75 HBV-related HCC patients who received curative surgical resection were analyzed to assess their association with HCC recurrence after surgery. The clinicopathological characteristics, which were significantly and independently associated with HCC recurrence, were identified, and were further combined with HBV pre-S2 gene deletion mutation to evaluate the performance of their combination in predicting HBV-related HCC recurrence.

## 2. Materials and Methods

### 2.1. Collection of Plasma Samples and Clinical Data of HCC Patients

The plasma samples were retrospectively collected from 75 HBV-related HCC patients who received curative surgical resection as monotherapy at the China Medical University Hospital (CMUH; Taichung, Taiwan) from March 2004 to September 2016 under the approval of the China Medical University & Hospital Research Ethics Committee (protocol no. CMUH107-REC1-080) and were stored at −80 °C until use. All patients included in this study were HBV-infected without concurrent HCV or HDV infection and did not experience virological failure after anti-viral treatment. The clinicopathological data were retrieved from the patient medical records of CMUH. All experiments were performed ethically according to the guidelines of the 1975 Declaration of Helsinki and written informed consent was obtained from all enrolled patients.

### 2.2. Detection of HBV Pre-S2 Gene Deletion Mutation in Plasma Samples

The pre-S2 gene deletion mutation was detected using an approach based on next-generation sequencing (NGS) analysis from the plasma samples of patients with HBV-related HCC as previously described [29]. Briefly, the HBV DNA was extracted from the plasma samples using the DNeasy Blood & Tissue kit (Qiagen, Valencia, CA, USA) and was then used as the template to amplify the entire pre-S gene region including pre-S1 and pre-S2 genes by two successive rounds of nested polymerase chain reaction (PCR) assay using pre-S gene-specific PCR primer sets and high-fidelity Platinum SuperFi DNA polymerase (Invitrogen, Carlsbad, CA, USA). The resulting pre-S gene PCR product was next analyzed by NGS using the NextSeq 500 system supplemented with the bcl2fastq Conversion Software v2.20 (Illumina, San Diego, CA, USA) according to the manufacturer’s instructions to measure the percentage of deletion mutations, which occur on the solely pre-S1 gene, solely pre-S2 gene, or both pre-S1 and pre-S2 genes. The presence of pre-S2 gene deletion mutation was defined by the percentage of deletion mutations on solely pre-S2 genes or both pre-S1 and pre-S2 genes above a cut-off of 4.643% in the plasma samples.

### 2.3. Statistical Analysis

The chi-square test was performed to assess the association between clinicopathological characteristics and HCC recurrence in patients. The Cox proportional-hazards regression model was conducted for the univariate and multivariate analyses of recurrence-free survival (RFS) of patients. The Kaplan-Meier method and the log-rank test were carried out to construct and compare the RFS curves of patients, respectively. The receiver operating characteristic (ROC) curves were plotted to compare the performance of clinicopathological characteristics in differentiating patients with HCC recurrence from patients without HCC recurrence and the Hanley-McNeil test was applied to determine the area under the ROC curve (AUC).

## 3. Results

### 3.1. Profile of Clinicopathological Characteristics of the 75 Enrolled Patients with HBV-Related HCC and Receiving Curative Surgical Resection in This Study

As shown in Table 1, of the total 75 HCC patients, 68 (91%) were men and 7 (9%) were women; 60 (80%) were infected with HBV genotype B and 15 (20%) were infected with HBV genotype C. The median age (range) in all patients was 53 (26 to 78) years and the median HBV DNA level (range) in serum was 2.1 × 10^4^ (21.5 to 1.5 × 10^8^) copies/mL. All 65 (100%) patients with available data were HBV surface antigen (HBsAg)-positive and 62 of 71 (87%) patients with available data were HBV e antigen (HBeAg)-negative in serum. The median tumor size (range) in all patients was 4.5 (1.1 to 19.5) cm. There were 40 (53%) patients with American Joint Committee on Cancer (AJCC) tumor-node-metastasis (TNM) stage I, 20 (27%) patients with TNM stage II, 7 (9%) patients with TNM stage IIIA, 5 (7%) patients with TNM stage IIIB, and 3 (4%) patients with TNM stage IIIC; however, there were no patients with TNM stage IVA or IVB. Moreover, by the NGS-based analysis, the presence of HBV pre-S2 gene deletion mutation in plasma was detected in 31 of 75 (41%) patients but not in the other 44 (59%) patients. After curative surgical resection, 52 of 75 (69%) patients suffered HCC recurrence but the other 23 (31%) patients did not; the median RFS (range) of all patients was 26.2 (1.5 to 158.9) months at a maximum follow-up of 158.9 months. In addition, the overall survival (OS) rate of patients after surgery was 79% with the median OS (range) of 61.5 (2.5 to 171.1) months at a maximum follow-up of 171.1 months.

### 3.2. Association of the Presence of HBV Pre-S2 Gene Deletion Mutation or AJCC TNM Stage IIIA-IIIC Alone with a Higher Risk of HCC Recurrence in Patients after Curative Surgical Resection

**Figure 1:**
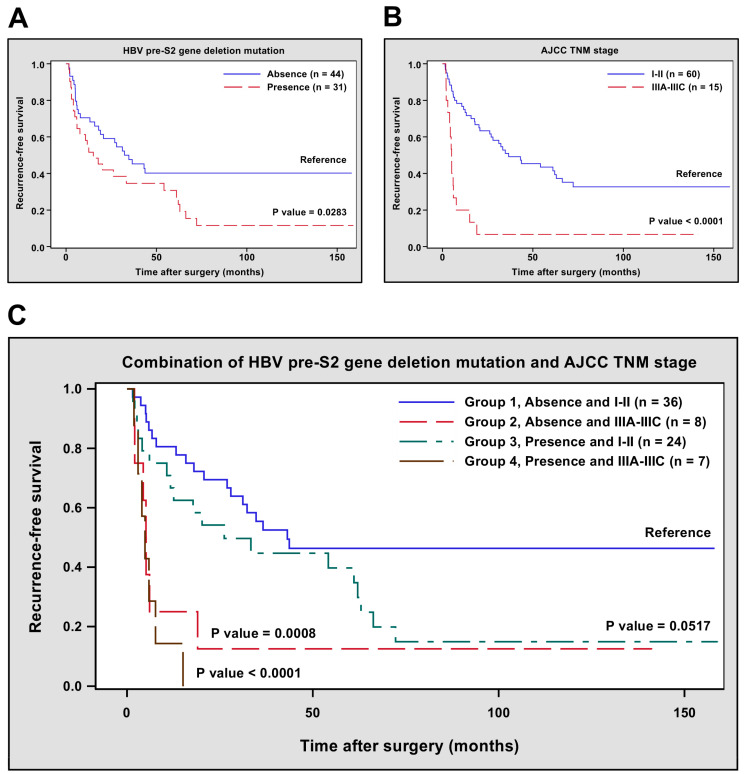
Kaplan-Meier curves for comparing the RFS of patients with different statuses of HBV pre-S2 gene deletion mutation and/or AJCC TNM stage after curative surgical resection. (**A**) RFS difference between patients with absence and presence of pre-S2 gene deletion mutation. (**B**) RFS difference between patients with AJCC TNM stage I–II and stage IIIA-IIIC. (**C**) RFS difference between patients with absence or presence of pre-S2 gene deletion mutation in combination with AJCC TNM stage I–II or stage IIIA-IIIC.

To identify the clinicopathological characteristics, which were associated with HBV-related HCC recurrence, the association between clinicopathological characteristics and HCC recurrence in the 75 HBV-related HCC patients after resection surgery was assessed by the chi-square test. As shown in Table 2, the presence of pre-S2 gene deletion mutation in plasma was significantly more frequent in patients with HCC recurrence (26 of 52 (50%) patients) than in patients without HCC recurrence (5 of 23 (22%) patients) (*p*-value = 0.0248). Among the other clinicopathological characteristics analyzed, AJCC TNM stage was the only factor which exhibited a significant association with HCC recurrence; patients with HCC recurrence had a significantly higher proportion of AJCC TNM stage IIIA-IIIC (14 of 52 (27%) patients) than patients without HCC recurrence did (1 of 23 (4%) patients) (*p*-value = 0.0286). Moreover, based on the analysis of Kaplan-Meier RFS curves, patients with the presence of pre-S2 gene deletion mutation or AJCC TNM stage IIIA-IIIC displayed significantly poorer RFS than patients with the absence of pre-S2 gene deletion mutation or AJCC TNM stage I–II did (median RFS (range), 15.1 (1.6 to 158.9) vs. 32.6 (1.5 to 158.1) months, *p* value = 0.0283; 5.1 (1.9 to 141.3) vs. 33.1 (1.5 to 158.9) months, *p* value < 0.0001, respectively) (Figure 1A,B). In the univariate analysis of RFS, the presence of pre-S2 gene deletion mutation, AJCC TNM stage IIIA-IIIC, and Child-Pugh cirrhosis score B–C were significantly associated with poorer RFS of patients, but the serum albumin level of >3.8 g/dL was significantly associated with better RFS of patients (hazard ratio (HR) (95% confidence interval (CI)), 1.825 (1.058 to 3.149), *p* value = 0.0307; 4.048 (2.123 to 7.719), *p* value < 0.0001; 2.189 (1.195 to 4.013), *p* value = 0.0112; 0.515 (0.288 to 0.923), *p* value = 0.0258, respectively); however, in the multivariate analysis of RFS, only the presence of pre-S2 gene deletion mutation and AJCC TNM stage IIIA-IIIC were validated as the independent prognostic factors which showed a significantly negative impact on RFS of patients (HR (95% CI), 1.910 (1.065 to 3.425), *p* value = 0.0300; 3.822 (1.920 to 7.607), *p* value = 0.0001, respectively) (Table 3).

### 3.3. Association of the Presence of HBV Pre-S2 Gene Deletion Mutation Combined with AJCC TNM Stage IIIA-IIIC with a Much Higher Risk of HCC Recurrence in Patients after Curative Surgical Resection

**Figure 2:**
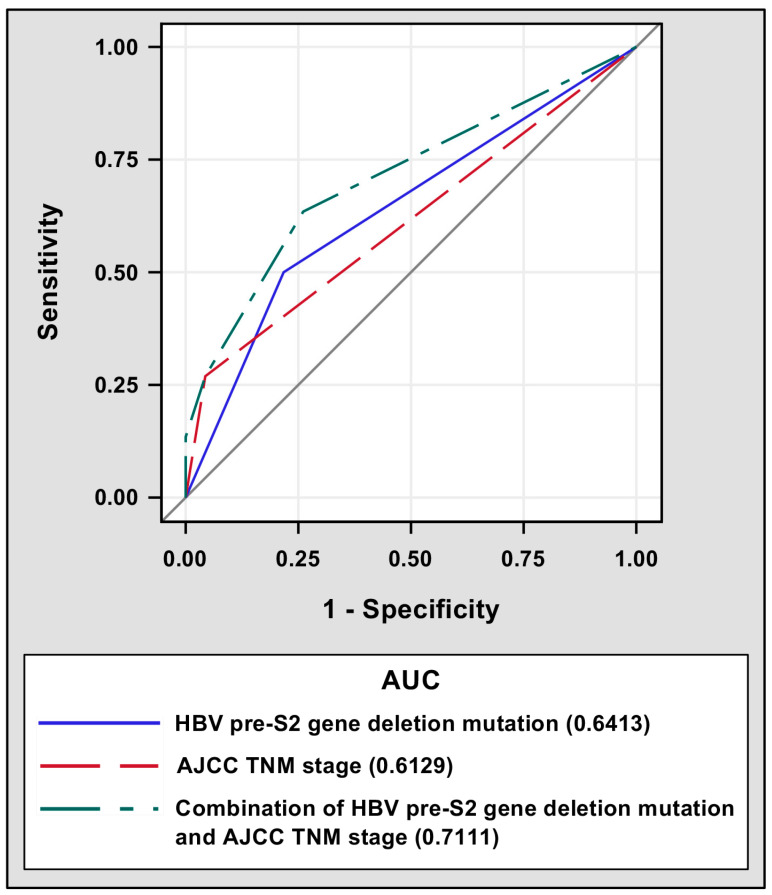
ROC curves for comparing the performance of HBV pre-S2 gene deletion mutation and/or AJCC TNM stage in predicting HCC recurrence after curative surgical resection. 52 patients with HCC recurrence and 23 patients without HCC recurrence were analyzed. The AUC for pre-S2 gene deletion mutation (solid blue line), AJCC TNM stage (dotted red line) or combination of both factors (dotted green line) was shown at the bottom of the plot.

To evaluate the prognostic performance of pre-S2 gene deletion mutation combined with AJCC TNM stage for HBV-related HCC recurrence after resection surgery, the 75 HBV-related HCC patients were divided into four groups according to the status of these two factors: Group 1, absence of pre-S2 gene deletion mutation and AJCC TNM stage I–II (36 patients); Group 2, absence of pre-S2 gene deletion mutation but AJCC TNM stage IIIA–IIIC (8 patients); Group 3, presence of pre-S2 gene deletion mutation but AJCC TNM stage I–II (24 patients); and Group 4, presence of pre-S2 gene deletion mutation and AJCC TNM stage IIIA–IIIC (7 patients). As shown by the Kaplan-Meier RFS analysis, the Group 2 and Group 4 patients, rather than the Group 3 patients, had significantly shorter RFS than the Group 1 patient did (median RFS (range), 5.1 (2.0 to 141.3) vs. 36.8 (1.5 to 158.1) months, *p* value = 0.0008; 4.8 (1.9 to 15.1) vs. 36.8 (1.5 to 158.1) months, *p* value < 0.0001, respectively) (Figure 1C). In the univariate analysis of RFS, the Group 2 and Group 4 combinations of pre-S2 gene deletion mutation and AJCC TNM stage were significantly associated with poorer RFS of patients (HR (95% CI), 4.071 (1.689 to 9.813), P value = 0.0018; 8.144 (3.179 to 20.864), *p* value < 0.0001, respectively); however, in the multivariate analysis of RFS, the Group 2, Group 3, and Group 4 combinations of pre-S2 gene deletion mutation and AJCC TNM stage displayed a significantly independent negative impact on RFS of patients (HR (95% CI), 4.522 (1.803 to 11.344), *p* value = 0.0013; 2.078 (1.068 to 4.043), *p* value = 0.0312; 6.660 (2.535 to 17.496), *p* value = 0.0001, respectively) (Table 4). Moreover, the ROC curve analysis verified that the combination of pre-S2 gene deletion mutation and AJCC TNM stage had the best performance in predicting HCC recurrence, as shown by the highest AUC (0.7111, 95% CI 0.6050 to 0.8173); in comparison, the pre-S2 gene deletion mutation alone had the second highest AUC (0.6413, 95% CI 0.5311 to 0.7515) and the AJCC TNM stage alone had the lowest AUC (0.6129, 95% CI 0.5386 to 0.6872) (Figure 2).

## 4. Discussion

Although curative resection surgery has been available for the treatment of HCC patients, an extremely high rate of HBV-related HCC recurrence after treatment is still a big challenge and leads to poor patient survival [30,31]. The presence of HBV pre-S2 gene deletion mutation in HCC patients has been recognized as an important and independent biomarker for predicting a higher recurrence risk of HBV-related HCC after curative resection therapy [25,26]. In this study, we found that patients who harbored pre-S2 gene deletion mutation in combination with AJCC TNM stage IIIA-IIIC exhibited a much higher recurrence risk of HCC than patients who harbored only one of each factor or none of both factors. Furthermore, the AJCC TNM stage was identified as a significant independent clinicopathological factor, which held the potential to be combined with pre-S2 gene deletion mutation as a combination biomarker with greater prognostic performance for predicting the recurrence of HBV-related HCC after surgical resection.

The prognostic value of the TNM stage combined with other clinicopathological factors in the prediction of HCC recurrence after curative surgical resection has been validated in several studies. Sonohara et al. showed that the established scoring system combining TNM stage and albumin-bilirubin grade (ALBI-T scoring) exhibited an independent prognostic performance for HCC recurrence [32]. Zhang et al. established a novel prognostic equation based on both TNM stage and tumor size and verified its independent performance in predicting HCC recurrence [33]. Liu et al. developed a novel prognostic nomogram consisting of the TNM stage and the expression signature of five tumor stage-related genes and ascertained its independent prediction potential for HCC recurrence [34]. Zhong et al. proposed a novel prognostic model based on the TNM stage together with the expression signature of four long noncoding RNAs (lncRNAs) and confirmed its capacity for independent prediction of HCC recurrence [35]. Fu et al. constructed a novel prognostic nomogram incorporating TNM stage, serum alpha-fetoprotein (AFP) level, vascular invasion, and the expression signature of 25 lncRNAs and validated its independent predictive potential for HCC recurrence [36]. Liu et al. built a prognostic scoring system comprising TNM stage, age, serum AFP level, resection margin, neutrophil/lymphocyte ratio, and aspartate aminotransferase (AST)/platelet ratio index and proved its independent predictive value for HCC recurrence [37]. Chen et al. showed that the combination of the TNM stage and the expression level of a glycophosphoprotein, osteopontin, displayed an independent prognostic significance in HCC recurrence [38]. However, the prognostic performance of the TNM stage in combination with the virological factors in HBV-related HCC patients for HCC recurrence after resection surgery remains largely undetermined. To our knowledge, this study is the first to discover that combination of TNM stage and HBV pre-S2 gene deletion mutation showed a better performance than either of these two factors alone in identifying patients with HBV-related HCC recurrence.

As an important HBV oncoprotein, pre-S2 mutant has been shown to predominantly accumulate in the endoplasmic reticulum (ER) of HBV-infected hepatocytes to induce ER stress and activate many ER stress-dependent or -independent oncogenic signaling pathways in promoting hepatocyte malignant transformation and eventually HCC development, including the signaling pathways related on oxidative DNA damage (calcium ions (Ca^2+^)/reactive oxygen species (ROS)), chromosome instability (Ca^2+^/μ-calpain/N-terminus truncated cyclin A), genomic instability (importin α1/Nijmegen breakage syndrome 1 (NBS1)), cell proliferation (vascular endothelial growth factor (VEGF)-A/VEGF receptor-2/protein kinase B (Akt)/mammalian target of the rapamycin (mTOR)), cell cycle progression (Jun activation domain-binding protein 1 (JAB1)/p27/cyclin-dependent kinase (Cdk2)/retinoblastoma protein (Rb)), cell growth (nuclear factor-κB (NF-κB)/p38 mitogen-activated protein kinase (p38 MAPK)/cyclooxygenase-2 (COX-2)), cell survival (B cell lymphoma-2 (Bcl-2)), aerobic glycolysis (Yin Yang 1 (YY1)/Myc/solute carrier family 2 (facilitated glucose transporter) member 1 (SLC2A1)), and lipid biosynthesis (sterol regulatory element-binding transcription factor 1 (SREBF1)/adenosine triphosphate citrate lyase (ACLY)/fatty acid desaturase 2 (FADS2)) [15,28,39]. However, the expression profile of these pre-S2 mutant-activated signaling pathways in tumor tissues of HBV-related HCC patients with the presence of pre-S2 gene deletion mutation combined with AJCC TNM stage IIIA-IIIC remains to be clarified. Considering that inhibition of the oncogenic signaling molecules activated by pre-S2 mutant has been shown to exert a chemopreventive effect on pre-S2 mutant-induced HCC tumor formation in a transgenic mouse model [17,18,19], to elucidate the molecular pathological mechanisms underlying the higher risk of HCC recurrence would be promising and important to develop novel preventive or therapeutic interventions for the treatment of this high-risk group of HCC patients after curative resection therapy following selection by the combination biomarker of pre-S2 gene deletion mutation and AJCC TNM stage. In addition, although the clinicopathological profile of the 75 HBV-related HCC patients enrolled in this study properly coincides with the representative profile of clinicopathological characteristics of a large patient population in Taiwan [40], further studies based on a large cohort of patients from multiple clinical centers are still required to validate the finding of this study.

## 5. Conclusions

This study proposed a combination of the HBV viral factor, pre-S2 gene deletion mutation, and the clinicopathological factor, AJCC TNM stage, as a novel combination biomarker with an improved performance in predicting HCC recurrence in HBV-related HCC patients after curative surgical resection.

## Figures and Tables

**Table 1:** Clinicopathological profile of 75 HBV-related HCC patients enrolled in this study.

Characteristics	No. of Patients (%) ^a^	Median (Range)
Age (years) ≤50 >50	75 (100) 27 (36) 48 (64)	53 (26–78) 43 (26–50) 60 (51–78)
Gender (women/men)	7 (9)/68 (91)	
Smoking (no/yes)	44 (59)/31 (41)	
Alcohol (no/yes)	46 (61)/29 (39)	
HBsAg (negative/positive/NA)	0 (0)/65 (100)/10	
HBeAg (negative/positive/NA)	62 (87)/9 (13)/4	
HBV genotype (B/C)	60 (80)/15 (20)	
HBV DNA (IU/mL) (20-1.7 × 10^8^/<20) ^b^ ≤1 × 10^4^ >1 × 10^4^	74 (100)/1 32 (43) 42 (57)	2.1 × 10^4^ (21.5–1.5 × 10^8^) ^d^ 8.4 × 10^2^ (21.5–9.3 × 10^3^) 4.3 × 10^5^ (1.2 × 10^4^–1.5 × 10^8^)
Albumin (g/dL) ≤3.8 >3.8	75 (100) 45 (60) 30 (40)	3.7 (1.2–4.9) 3.3 (1.2–3.8) 4.2 (3.9–4.9)
AST (U/L) ≤34 >34	75 (100) 14 (19) 61 (81)	60 (14–1052) 27 (14–34) 79 (35–1052)
ALT (U/L) ≤40 >40	75 (100) 25 (33) 50 (67)	55 (13–1338) 31 (13–40) 96.5 (41–1338)
AFP (ng/mL) (≤54,000/>54,000) ^c^ ≤400 >400	71 (100)/4 47 (66) 28 (34)	26.7 (1.8–36,600.0) ^e^ 13.8 (1.8–271.0) 1920 (461.7–36,600.0)
Tumor size (cm) ≤5 >5	75 (100) 38 (51) 37 (49)	4.5 (1.1–19.5) 2.4 (1.1–4.5) 10.0 (5.5–19.5)
Tumor encapsulation (no/yes/NA)	20 (32)/42 (68)/13	
Lymph node involvement (no/yes)	67 (89)/8 (11)	
Portal vein thrombosis (no/yes)	70 (93)/5 (7)	
Vascular invasion (no/yes)	48 (64)/27 (36)	
Distant metastasis (no/yes)	67 (89)/8 (11)	
Steatosis grade (0/1/2/3/NA)	14 (56)/10 (40)/1 (4)/0 (0)/50	
Metavir inflammation score (0/1/2/3/NA)	4 (9)/35 (80)/5 (11)/0 (0)/31	
Ishak fibrosis score (0/1/2/3/4/5/6/NA)	5 (9)/13 (23)/12 (22)/8 (14)/3 (5)/4 (7)/11 (20)/19	
Child-Pugh cirrhosis score (A/B/C)	57 (76)/16 (21)/2 (3)	
CLIP score (0/1/2/3/4/5/6)	33 (44)/23 (31)/10 (13)/8 (11)/1 (1)/0 (0)/0 (0)	
Tumor differentiation grade (1/2/3/4)	2 (3)/36 (48)/36 (48)/1 (1)	
BCLC stage (A/B/C/D)	38 (51)/29 (39)/7 (9)/1 (1)	
AJCC TNM stage (I/II/IIIA/IIIB/IIIC/IVA/IVB)	40 (53)/20 (27)/7 (9)/5 (7)/3 (4)/0 (0)/0 (0)	
HBV pre-S2 gene deletion mutation (absence/presence)	44 (59)/31 (41)	
HCC recurrence after surgery (month) ^f^ No Yes	75 (100) 23 (31) 52 (69)	26.2 (1.5–158.9) 141.3 (25.4–158.9) 11.2 (1.5–72.3)

^a^ Only patients with available data or with data within the detection range were counted to determine the percentage. ^b^ The detection range of HBV DNA level was 20 to 1.7 × 10^8^ IU/mL. ^c^ The highest detection limit of AFP level was 54,000 ng/mL. ^d,e^ Only data that falls within the detection range were analyzed. ^f^ Shown was recurrence-free survival time after curative surgical resection in the follow-up period. Abbreviations: HBV, hepatitis B virus; HCC, hepatocellular carcinoma; HBsAg, hepatitis B s antigen; HBeAg, hepatitis B e antigen; NA, not available; AST, aspartate aminotransferase; ALT, alanine aminotransferase; AFP, alpha-fetoprotein; CLIP, Cancer of the Liver Italian Program; BCLC, Barcelona Clinic Liver Cancer; AJCC, American Joint Committee on Cancer; TNM, tumor-node-metastasis.

**Table 2:** Clinicopathological association of HCC recurrence in 75 HBV-related HCC patients after surgery.

Characteristics ^a^	Non-Recurrence (No. of Patients (%))	Recurrence (No. of Patients (%))	*p* Value ^b^
Age (years) ≤50 >50	23 (100) 8 (35) 15 (65)	52 (100) 17 (33) 35 (67)	1.0000
Gender Women Men	23 (100) 2 (9) 21 (91)	52 (100) 5 (10) 47 (90)	1.0000
Smoking No Yes	23 (100) 11 (48) 12 (52)	52 (100) 33 (63) 19 (37)	0.2171
Alcohol No Yes	23 (100) 11 (48) 12 (52)	52 (100) 35 (67) 17 (33)	0.1291
HBsAg ^c^ Negative Positive	23 (100) 0 (0) 23 (100)	52 (100) 0 (0) 52 (100)	
HBeAg Positive Negative	23 (100) 3 (17) 19 (83)	52 (100) 6 (17) 43 (83)	1.0000
HBV genotype B C	23 (100) 20 (87) 3 (13)	52 (100) 40 (77) 12 (23)	0.3690
HBV DNA (copies/mL) ≤1 × 10^4^ >1 × 10^4^	23 (100) 12 (52) 11 (48)	51 (100) 20 (38) 31 (62)	0.3219
Albumin (g/dL) ≤3.8 >3.8	23 (100) 11 (48) 12 (52)	52 (100) 35 (67) 17 (33)	0.1291
AST (U/L) ≤34 >34	23 (100) 3 (13) 20 (87)	52 (100) 11 (21) 41 (79)	0.5293
ALT (U/L) ≤40 >40	23 (100) 5 (22) 18 (78)	52 (100) 20 (38) 32 (62)	0.1914
AFP (ng/mL) ≤400 >400	23 (100) 15 (65) 8 (35)	52 (100) 32 (62) 20 (38)	0.8017
Tumor size (cm) ≤5 >5	23 (100) 12 (52) 11 (48)	52 (100) 26 (50) 26 (50)	0.9965
Tumor encapsulation No Yes	20 (100) 6 (30) 14 (70)	42 (100) 14 (33) 28 (67)	1.0000
Lymph node involvement No Yes	23 (100) 18 (78) 5 (22)	52 (100) 49 (94) 3 (6)	0.0528
Portal vein thrombosis No Yes	23 (100) 22 (96) 1 (4)	52 (100) 48 (92) 4 (8)	0.9924
Vascular invasion No Yes	23 (100) 17 (74) 6 (26)	52 (100) 31 (60) 21 (40)	0.3009
Distant metastasis No Yes	23 (100) 22 (96) 1 (4)	52 (100) 45 (87) 7 (13)	0.4219
Steatosis grade ^d^ 0–1 2	7 (100) 7 (100) 0 (0)	18 (100) 17 (94) 1 (6)	1.0000
Metavir inflammation score ^e^ 0–1 2	11 (100) 10 (91) 1 (9)	33 (100) 29 (88) 4 (12)	1.0000
Ishak fibrosis score 0–3 4–6	13 (100) 10 (77) 3 (23)	43 (100) 28 (65) 15 (35)	0.5144
Child-Pugh cirrhosis score A B–C	23 (100) 20 (87) 3 (23)	52 (100) 37 (71) 15 (29)	0.2399
CLIP score ^f^ 0–3 4	23 (100) 23 (100) 0 (0)	52 (100) 51 (98) 1 (2)	1.0000
Tumor differentiation grade 1–2 3–4	23 (100) 13 (57) 10 (43)	52 (100) 25 (48) 27 (52)	0.6181
BCLC stage A–B C–D	23 (100) 22 (96) 1 (4)	52 (100) 45 (87) 7 (13)	0.4219
AJCC TNM stage ^g^ I–II IIIA–IIIC	23 (100) 22 (96) 1 (4)	52 (100) 38 (73) 14 (27)	0.0286 *
HBV pre-S2 gene deletion mutation Absence Presence	23 (100) 18 (78) 5 (22)	52 (100) 26 (50) 26 (50)	0.0248 *

^a^ Only patients with available data were included in the statistical analysis. ^b^ P value was determined by the chi-square test. ^c^ No patients were HBsAg negative for analysis. ^d^ No patients were steatosis grade 3 for analysis. ^e^ No patients were Metavir inflammation score 3 for analysis. ^f^ No patients were CLIP score 5–6 for analysis. ^g^ No patients were AJCC TNM stage IVA-IVB for analysis. * *p* value < 0.05. Abbreviations: HCC, hepatocellular carcinoma; HBV, hepatitis B virus; HBsAg, hepatitis B s antigen; HBeAg, hepatitis B e antigen; AST, aspartate aminotransferase; ALT, alanine aminotransferase; AFP, alpha-fetoprotein; CLIP, Cancer of the Liver Italian Program; BCLC, Barcelona Clinic Liver Cancer; AJCC, American Joint Committee on Cancer; TNM, tumor-node-metastasis.

**Table 3:** Univariate and multivariate RFS analyses of clinicopathological characteristics in 75 HBV-related HCC patients.

Characteristics ^a^	Univariate Analysis			Multivariate Analysis		
	HR	95% CI	*p* Value ^b^	HR	95% CI	*p* Value ^b^
Age (years) ≤50 >50	Reference 0.951	0.532–1.700	0.8666			
Gender Women Men	Reference 1.043	0.414–2.627	0.9284			
Smoking No Yes	Reference 0.886	0.503–1.560	0.6750			
Alcohol No Yes	Reference 0.884	0.494–1.580	0.6773			
HBsAg ^c^ Negative Positive						
HBeAg Negative Positive	Reference 1.234	0.523–2.910	0.6307			
HBV genotype C B	Reference 0.583	0.304–1.117	0.1040			
HBV DNA (IU/mL) ≤1 × 10^4^ >1 × 10^4^	Reference 1.645	0.934–2.895	0.0846			
Albumin (g/dL) ≤3.8 >3.8	Reference 0.515	0.288–0.923	0.0258 *	Reference 0.658	0.349–1.241	0.1960
AST (U/L) ≤34 >34	Reference 0.865	0.444–1.684	0.6691			
ALT (U/L) ≤40 >40	Reference 0.797	0.456–1.394	0.4267			
AFP (ng/mL) ≤400 >400	Reference 1.305	0.745–2.285	0.3524			
Tumor size (cm) ≤5 >5	Reference 1.490	0.863–2.572	0.1525			
Tumor encapsulation No Yes	Reference 0.901	0.474–1.713	0.7508			
Lymph node involvement No Yes	Reference 0.333	0.104–1.071	0.0652			
Portal vein thrombosis No Yes	Reference 1.668	0.600–4.633	0.3264			
Vascular invasion No Yes	Reference 1.677	0.962–2.924	0.0681			
Distant metastasis No Yes	Reference 2.259	0.999–5.101	0.0502			
Steatosis grade ^d^ 0–1 2	Reference 3.473	0.418–28.879	0.2493			
Metavir inflammation score ^e^ 0–1 2	Reference 0.731	0.256–2.088	0.5583			
Ishak fibrosis score 0–3 4–6	Reference 1.261	0.670–2.373	0.4714			
Child-Pugh cirrhosis score A B–C	Reference 2.189	1.195–4.013	0.0112 *	Reference 1.876	0.957–3.676	0.0668
CLIP score ^f^ 0–3 4	Reference 2.426	0.328–17.911	0.3850			
Tumor differentiation grade 1–2 3–4	Reference 1.246	0.722–2.150	0.4288			
BCLC stage A–B C–D	Reference 1.927	0.867–4.284	0.1077			
AJCC TNM stage ^g^ I–II IIIA–IIIC	Reference 4.048	2.123–7.719	<0.0001 ***	Reference 3.822	1.920–7.607	0.0001 ***
HBV pre-S2 gene deletion mutation Absence Presence	Reference 1.825	1.058–3.149	0.0307 *	Reference 1.910	1.065–3.425	0.0300 *

^a^ Only patients with available data were included in the statistical analysis. ^b^ *p* value was determined by the Cox proportional-hazards regression model. ^c^ No patients were HBsAg negative for analysis. ^d^ No patients were steatosis grade 3 for analysis. ^e^ No patients were Metavir inflammation score 3 for analysis. ^f^ No patients were CLIP score 5-6 for analysis. ^g^ No patients were AJCC TNM stage IVA-IVB for analysis. * *p* value < 0.05; *** *p* value < 0.001. Abbreviations: RFS, recurrence-free survival; HBV, hepatitis B virus; HCC, hepatocellular carcinoma; HR, hazard ratio; CI, confidence interval; HBsAg, hepatitis B s antigen; HBeAg, hepatitis B e antigen; AST, aspartate aminotransferase; ALT, alanine aminotransferase; AFP, alpha-fetoprotein; CLIP, Cancer of the Liver Italian Program; BCLC, Barcelona Clinic Liver Cancer; AJCC, American Joint Committee on Cancer; TNM, tumor-node-metastasis.

**Table 4:** Univariate and multivariate RFS analyses of HBV pre-S2 gene deletion mutation combined with AJCC TNM stage in 75 HBV-related HCC patients.

Characteristics	Univariate Analysis			Multivariate Analysis		
	HR	95% CI	*p* Value ^a^	HR	95% CI	*p* Value ^a^
Albumin (g/dL) ≤3.8 >3.8	Reference 0.515	0.288–0.923	0.0258 *	Reference 0.658 0.630	0.349–1.241 0.328–1.210	0.1960 ^c^ 0.1649 ^d^
Child-Pugh cirrhosis score A B–C	Reference 2.189	1.195–4.013	0.0112 *	Reference 1.876 1.901	0.957–3.676 0.970–3.725	0.0668 ^c^ 0.0612 ^d^
AJCC TNM stage ^b^ I–II IIIA–IIIC	Reference 4.048	2.123–7.719	<0.0001 ***	Reference 3.822	1.920–7.607	0.0001 ***^,c^
HBV pre-S2 gene deletion mutation Absence Presence	Reference 1.825	1.058–3.149	0.0307 *	Reference 1.910	1.065–3.425	0.0300 *^,c^
Combination of HBV pre-S2 gene deletion mutation and AJCC TNM stage Group 1, Absence and I–II Group 2, Absence and IIIA–IIIC Group 3, Presence and I–II Group 4, Presence and IIIA–IIIC	Reference 4.071 1.854 8.144	1.689–9.813 0.980–3.505 3.179–20.864	0.0018 ** 0.0576 <0.0001 ***	Reference 4.522 2.078 6.660	1.803–11.344 1.068–4.043 2.535–17.496	0.0013 **^,d^ 0.0312 *^,d^ 0.0001 ***^,d^

^a^ *p* value was determined by the Cox proportional-hazards regression model. ^b^ No patients were AJCC TNM stage IVA-IVB for analysis. ^c,d^ Multivariate analysis was performed between the characteristics labeled with the same symbols. Abbreviations: RFS, recurrence-free survival; HBV, hepatitis B virus; HCC, hepatocellular carcinoma; TNM, tumor-node-metastasis; AJCC, American Joint Committee on Cancer. * *p* value < 0.05; ** *p* value < 0.01; *** *p* value < 0.001.

## Data Availability

The data presented in this study are available in the article.

## References

[1] Venook A.P., Papandreou C., Furuse J., de Guevara L.L. (2010). The Incidence and Epidemiology of Hepatocellular Carcinoma: A Global and Regional Perspective. Oncologist.

[2] Cheng K.-C., Lin W.-Y., Liu C.-S., Lin C.-C., Lai H.-C., Lai S.-W. (2016). Association of different types of liver disease with demographic and clinical factors. Biomed. Pharmacother..

[3] Wall W.J., Marotta P.J. (2000). Surgery and transplantation for hepatocellular cancer. Liver Transplant..

[4] Marín-Hargreaves G., Azoulay D., Bismuth H. (2003). Hepatocellular carcinoma: Surgical indications and results. Crit. Rev. Oncol..

[5] Kanda T., Ogasawara S., Chiba T., Haga Y., Omata M., Yokosuka O. (2015). Current management of patients with hepatocellular carcinoma. World J. Hepatol..

[6] Makary M.S., Khandpur U., Cloyd J.M., Mumtaz K., Dowell J.D. (2020). Locoregional Therapy Approaches for Hepatocellular Carcinoma: Recent Advances and Management Strategies. Cancers.

[7] Llovet J.M., Bruix J. (2008). Novel advancements in the management of hepatocellular carcinoma in 2008. J. Hepatol..

[8] Llovet J.M., Ricci S., Mazzaferro V., Hilgard P., Gane E., Blanc J.F., de Oliveira A.C., Santoro A., Raoul J.L., Forner A. (2008). Sorafenib in advanced hepatocellular carcinoma. N. Engl. J. Med..

[9] Beasley R.P., Hwang L.Y. (1984). Hepatocellular carcinoma and hepatitis B virus. Semin. Liver Dis..

[10] Bosetti C., Turati F., La Vecchia C. (2014). Hepatocellular carcinoma epidemiology. Best Pr. Res. Clin. Gastroenterol..

[11] Seeger C., Mason W.S. (2000). Hepatitis B Virus Biology. Microbiol. Mol. Biol. Rev..

[12] Tsukuda S., Watashi K. (2020). Hepatitis B virus biology and life cycle. Antivir. Res..

[13] Wang H.-C., Wu H.-C., Chen C.-F., Fausto N., Lei H.-Y., Su I.-J. (2003). Different Types of Ground Glass Hepatocytes in Chronic Hepatitis B Virus Infection Contain Specific Pre-S Mutants that May Induce Endoplasmic Reticulum Stress. Am. J. Pathol..

[14] Su I.-J., Wang H.-C., Wu H.C., Huang W.-Y. (2008). Ground glass hepatocytes contain pre-S mutants and represent preneoplastic lesions in chronic hepatitis B virus infection. J. Gastroenterol. Hepatol..

[15] Teng C.-F., Wu H.C., Shyu W.-C., Jeng L.-B., Su I.-J. (2017). Pre-S2 Mutant-Induced Mammalian Target of Rapamycin Signal Pathways as Potential Therapeutic Targets for Hepatitis B Virus-Associated Hepatocellular Carcinoma. Cell Transplant..

[16] Teng Y.-C., Neo J.C., Wu J.-C., Chen Y.-F., Kao C.-H., Tsai T.-F. (2017). Expression of a hepatitis B virus pre-S2 deletion mutant in the liver results in hepatomegaly and hepatocellular carcinoma in mice. J. Pathol..

[17] Hsieh Y.-H., Su I.-J., Yen C.-J., Tsai T.-F., Tsai H.-W., Tsai H.-N., Huang Y.-J., Chen Y.-Y., Ai Y.-L., Kao L.-Y. (2013). Histone deacetylase inhibitor suberoylanilide hydroxamic acid suppresses the pro-oncogenic effects induced by hepatitis B virus pre-S 2 mutant oncoprotein and represents a potential chemopreventive agent in high-risk chronic HBV patients. Carcinogenesis.

[18] Teng C.-F., Hsieh W.-C., Wu H.C., Lin Y.-J., Tsai H.-W., Huang W., Su I.-J. (2015). Hepatitis B Virus Pre-S2 Mutant Induces Aerobic Glycolysis through Mammalian Target of Rapamycin Signal Cascade. PLoS ONE.

[19] Teng C.-F., Yu C.-H., Chang H.-Y., Hsieh W.-C., Wu T.-H., Lin J.-H., Wu H.-C., Jeng L.-B., Su I.-J. (2019). Chemopreventive Effect of Phytosomal Curcumin on Hepatitis B Virus-Related Hepatocellular Carcinoma in A Transgenic Mouse Model. Sci. Rep..

[20] Chen C.H., Hung C.H., Lee C.M., Hu T.H., Wang J.H., Wang J.C., Lu S.N., Changchien C.S. (2007). Pre-S deletion and complex mutations of hepatitis B virus related to advanced liver disease in HBeAg-negative patients. Gastroenterology.

[21] Shen F.-C., Su I.-J., Wu H.-C., Hsieh Y.-H., Yao W.-J., Young K.-C., Chang T.-C., Hsieh H.-C., Tsai H.-N., Huang W. (2009). A pre-S gene chip to detect pre-S deletions in hepatitis B virus large surface antigen as a predictive marker for hepatoma risk in chronic hepatitis B virus carriers. J. Biomed. Sci..

[22] Sinn D.H., Choi M.S., Gwak G.-Y., Paik Y.-H., Lee J.H., Koh K.C., Paik S.W., Yoo B.C. (2013). Pre-S Mutation Is a Significant Risk Factor for Hepatocellular Carcinoma Development: A Long-Term Retrospective Cohort Study. Dig. Dis. Sci..

[23] Tsai H.-W., Lin Y.-J., Lin P.-W., Wu H.-C., Hsu K.-H., Yen C.-J., Chan S.-H., Huang W., Su I.-J. (2011). A clustered ground-glass hepatocyte pattern represents a new prognostic marker for the recurrence of hepatocellular carcinoma after surgery. Cancer.

[24] Tsai H.-W., Lin Y.-J., Wu H.-C., Chang T.-T., Wu I.-C., Cheng P.-N., Yen C.-J., Chan S.-H., Huang W., Su I.-J. (2016). Resistance of ground glass hepatocytes to oral antivirals in chronic hepatitis B patients and implication for the development of hepatocellular carcinoma. Oncotarget.

[25] Yen C.-J., Ai Y.-L., Tsai H.-W., Chan S.-H., Cheng K.-H., Lee Y.-P., Kao C.-W., Wang Y.-C., Chen Y.-L., Lin C.-H. (2018). Hepatitis B virus surface gene pre-S_2_mutant as a high-risk serum marker for hepatoma recurrence after curative hepatic resection. Hepatology.

[26] Teng C.-F., Li T.-C., Huang H.-Y., Lin J.-H., Chen W.-S., Shyu W.-C., Wu H.-C., Peng C.-Y., Su I.-J., Jeng L.-B. (2020). Next-Generation Sequencing-Based Quantitative Detection of Hepatitis B Virus Pre-S Mutants in Plasma Predicts Hepatocellular Carcinoma Recurrence. Viruses.

[27] Teng C.-F., Li T.-C., Huang H.-Y., Chan W.-L., Wu H.-C., Shyu W.-C., Su I.-J., Jeng L.-B. (2020). Hepatitis B virus pre-S2 deletion (nucleotide 1 to 54) in plasma predicts recurrence of hepatocellular carcinoma after curative surgical resection. PLoS ONE.

[28] Teng C.-F., Wu H.-C., Su I.-J., Jeng L.-B. (2020). Hepatitis B Virus Pre-S Mutants as Biomarkers and Targets for the Development and Recurrence of Hepatocellular Carcinoma. Viruses.

[29] Teng C.-F., Huang H.-Y., Li T.-C., Shyu W.-C., Wu H.-C., Lin C.-Y., Su I.-J., Jeng L.-B. (2018). A Next-Generation Sequencing-Based Platform for Quantitative Detection of Hepatitis B Virus Pre-S Mutants in Plasma of Hepatocellular Carcinoma Patients. Sci. Rep..

[30] Kubo S., Takemura S., Tanaka S., Shinkawa H., Nishioka T., Nozawa A., Kinoshita M., Hamano G., Ito T., Urata Y. (2015). Management of hepatitis B virus infection during treatment for hepatitis B virus-related hepatocellular carcinoma. World J. Gastroenterol..

[31] Zamor P.J., Delemos A.S., Russo M.W. (2017). Viral hepatitis and hepatocellular carcinoma: Etiology and management. J. Gastrointest. Oncol..

[32] Sonohara F., Yamada S., Tanaka N., Suenaga M., Takami H., Hayashi M., Niwa Y., Sugimoto H., Hattori N., Kanda M. (2019). Perioperative and prognostic implication of albumin-bilirubin-TNM score in Child-Pugh class A hepatocellular carcinoma. Ann. Gastroenterol. Surg..

[33] Zhang Y., Chen S.-W., Liu L.-L., Yang X., Cai S.-H., Yun J.-P. (2018). A model combining TNM stage and tumor size shows utility in predicting recurrence among patients with hepatocellular carcinoma after resection. Cancer Manag. Res..

[34] Liu H., Yan Y., Chen R., Zhu M., Lin J., He C., Shi B., Wen K., Mao K., Xiao Z. (2020). Integrated nomogram based on five stage-related genes and TNM stage to predict 1-year recurrence in hepatocellular carcinoma. Cancer Cell Int..

[35] Zhong Y., Yang Y., He L., Zhou Y., Cheng N., Chen G., Zhao B., Wang Y., Wang G., Liu X. (2021). Development of Prognostic Evaluation Model to Predict the Overall Survival and Early Recurrence of Hepatocellular Carcinoma. J. Hepatocell. Carcinoma.

[36] Fu Y., Wei X., Han Q., Le J., Ma Y., Lin X., Xu Y., Liu N., Wang X., Kong X. (2021). Identification and characterization of a 25-lncRNA prognostic signature for early recurrence in hepatocellular carcinoma. BMC Cancer.

[37] Liu Y., Wang Z.-X., Cao Y., Zhang G., Chen W.-B., Jiang C.-P. (2016). Preoperative inflammation-based markers predict early and late recurrence of hepatocellular carcinoma after curative hepatectomy. Hepatobiliary Pancreat. Dis. Int..

[38] Chen R.-X., Xia Y.-H., Cui J.-F., Xue T.-C., Ye S.-L. (2010). Osteopontin, a single marker for predicting the prognosis of patients with tumor-node-metastasis stage I hepatocellular carcinoma after surgical resection. J. Gastroenterol. Hepatol..

[39] Lin Y.-T., Jeng L.-B., Chan W.-L., Su I.-J., Teng C.-F. (2021). Hepatitis B Virus Pre-S Gene Deletions and Pre-S Deleted Proteins: Clinical and Molecular Implications in Hepatocellular Carcinoma. Viruses.

[40] Liu P.-H., Hsu C.-Y., Hsia C.-Y., Lee Y.-H., Su C.-W., Huang Y.-H., Lee F.-Y., Lin H.-C., Huo T.-I. (2016). Prognosis of hepatocellular carcinoma: Assessment of eleven staging systems. J. Hepatol..

